# Editorial for the Special Issue “Gel Formation Processes and Materials for Functional Thin Films (1st Edition)”

**DOI:** 10.3390/gels12070568

**Published:** 2026-06-27

**Authors:** Denitsa Elenkova, Katerina Lazarova

**Affiliations:** 1 Faculty of Chemistry and Pharmacy, Sofia University “St. Kliment Ohridski”, 1 James Boucher Blvd., 1164 Sofia, Bulgaria; 2 Institute of Optical Materials and Technologies ‘‘Acad. J. Malinowski’’, Bulgarian Academy of Sciences, Akad. G. Bonchev Str., Bl. 109, 1113 Sofia, Bulgaria

## 1. Introduction

In recent years, gel-based materials have evolved from being considered soft, equilibrium-limited systems to highly tunable functional platforms capable of operating under non-equilibrium conditions and external stimulation [1,2,3]. Their unique combination of water-rich microstructure, mechanical softness, and molecular adaptability has positioned them at the core of emerging technologies in bioelectronics, sensing, energy storage, and environmental remediation [4,5,6].

Particularly important is the shift toward engineering gels not only as passive matrices but as active, responsive components whose structural and transport properties can be precisely controlled through molecular design, supramolecular interactions, and processing history [7,8,9,10]. This includes, for example, the use of peptide self-assembly to create ordered nanostructures, polymer-grafted architectures for stimuli-responsive thin films, and hybrid organic–inorganic networks for multifunctional devices [11,12,13,14].

Recent advances in hydrogel engineering and soft functional materials clearly demonstrate that properties such as swelling, conductivity, mechanical response, and optical behavior can now be rationally programmed through molecular and structural design rather than optimized solely through empirical approaches [14,15,16,17]. At the same time, the integration of gels into thin-film architectures has significantly expanded their application space, particularly in sensing, bioelectronics, and energy-storage technologies [5,18,19].

Within this context, this Special Issue brings together recent contributions that explore the formation, structure–property relationships, and application potential of gelated thin-film systems and related gel-derived functional materials across a broad range of material platforms and functional targets.

## 2. Statistical Overview of This Special Issue

**Figure 1:**
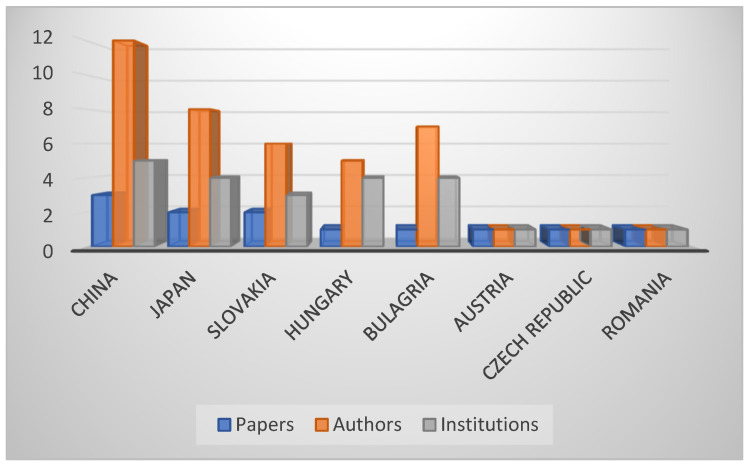
Countries represented in this Special Issue and their associated article contributions, authors, and research institutions.

This Special Issue comprises eight research articles authored by 41 researchers affiliated with 20 universities and scientific institutions from eight countries: Bulgaria, China, Japan, Hungary, Slovakia, Austria, the Czech Republic, and Romania. Among the published contributions, one article represents a particularly broad international collaboration involving researchers from five different countries. Figure 1 summarizes the distribution of articles by country, together with the numbers of participating institutions and contributing authors.

The contributions can be broadly grouped into several thematic areas. Three papers focus on porous inorganic and ceramic materials, including ordered mesoporous silica, uniform-pore alumina ceramics, and hierarchical silicon nitride aerogels. Two studies employ agarose-based platforms for neuronal growth control and neural circuit engineering. Additional contributions address flexible supercapacitor electrodes, multilayer ionogel sensors for soft electronics, and humidity-responsive polymer thin films. Together, these topics demonstrate the remarkable breadth of current gel research and illustrate how gel-derived materials continue to bridge fundamental science and advanced technological applications.

A total of 41 authors from 20 universities and research institutions contributed to this Special Issue. The published papers reflect a diverse range of collaboration patterns, from single-author studies to multinational research teams involving institutions from several countries.

Overall, this Special Issue demonstrates a balanced combination of inorganic, hybrid, and polymer-based gel systems, with a strong focus on structure–property relationships and functional performance in thin-film architectures.

## 3. Contribution Overview of This Special Issue

The papers collected in this Special Issue illustrate the wide range of scientific and technological opportunities offered by gel-based materials, spanning porous inorganic systems, advanced ceramic aerogels, bioengineered gel platforms, flexible sensing devices, energy-storage materials, and functional thin films. Despite the diversity of applications, a common theme emerges throughout the issue: the deliberate control of gel structure across multiple length scales in order to tailor transport phenomena, mechanical properties, biological interactions, and device performance.

Several contributions focus on the design of porous inorganic materials obtained through templating and sol–gel-related approaches. One study demonstrates the synthesis of ordered mesoporous silica using urea-based cationic gemini surfactants, revealing how surfactant molecular structure influences particle morphology, lattice parameters, and pore ordering, thereby providing insight into structure–property relationships in mesoporous silica systems. A related contribution reports the fabrication of uniform-pore η-Al_2_O_3_ ceramics through microemulsion-assisted sol–gel processing. By employing kinetically stable CTAB/hexanol/water microemulsions as soft templates, the authors achieve highly uniform mesopores, high specific surface area, and low density, highlighting the potential of scalable templating approaches for porous ceramic production. Hierarchical structural design is further explored in a study describing Si_3_N_4_ nanoparticle-reinforced Si_3_N_4_ nanofiber aerogels, where gradient pore architectures combine low density with mechanical robustness, thermal insulation, and electromagnetic-wave transparency, demonstrating the multifunctionality achievable in advanced ceramic gel-derived materials.

Energy-related applications are represented by a flexible CNFs/ZIF-8/PANI composite membrane prepared through interfacial growth and partial metal–organic framework etching. The resulting hierarchical micro/mesoporous structure enhances ion transport and electrochemical accessibility, enabling high specific capacitance and attractive performance in symmetric supercapacitor devices. The work illustrates how gel-based composite architectures can simultaneously address mechanical flexibility and electrochemical efficiency.

This issue also highlights the growing importance of gel materials in bioengineering and neuroengineering. Two complementary studies employ dynamically modifiable agarose gel platforms to investigate neuronal development and circuit formation. In one case, precise control of neurite elongation enables quantitative analysis of axon specification, revealing critical and definitive neurite-length thresholds that govern neuronal polarization. In the other, real-time infrared laser microfabrication is used to construct complex neuronal wiring geometries, including neurite crossings, while maintaining long-term culture stability through glial co-culture support. Together, these studies demonstrate how adaptable gel environments can serve as powerful tools for studying and engineering neural systems.

Soft functional materials are further represented by multilayer ionogel systems developed for distributed sensing applications. By combining a pressure-responsive ionic-liquid sensing layer with a positioning layer, the authors create flexible devices capable of simultaneously detecting pressure and touch location. Experimental characterization together with constitutive and equivalent-circuit modeling establishes a foundation for the integration of ionogel materials into future soft robotic and flexible electronic systems.

The final contribution investigates humidity-responsive thin gel films based on PVA derivatives containing grafted PDMA chains with different macromolecular architectures. The study demonstrates how thermal treatment influences film densification, water uptake, swelling behavior, hysteresis, optical response, and sensing performance. The results show that careful optimization of grafting architecture and annealing temperature enables high-resolution humidity detection, while also revealing the potential of these materials for colorimetric optical sensing. This work highlights the importance of processing–structure–property relationships in the development of functional polymer gel coatings.

Taken together, the studies collected in this Special Issue demonstrate how precise control over gel structure and processing can be exploited to achieve targeted functionalities. The reported advances include hierarchical porous architectures for electrochemical energy storage, ordered mesoporous and ceramic materials prepared by sol–gel routes, agarose-based platforms for neuronal network engineering, flexible ionogel sensors, thermally insulating ceramic aerogels, and polymer thin films with tunable humidity-sensing performance. These examples highlight the broad range of material systems and functional approaches currently driving progress in gel-based thin-film technologies.

## 4. Conclusions

The contributions in this Special Issue demonstrate the versatility of gel-based materials across inorganic, polymer, and hybrid systems, covering applications in energy-storage, sensing, environmental technologies, and biomedical and neuroengineering platforms. The studies collectively show how sol–gel processing, polymer architecture, and structural design at the micro- and nanoscale govern key functional properties such as porosity, transport behavior, mechanical response, and environmental sensitivity. Overall, this Special Issue highlights the central role of controlled gel formation and processing in enabling next-generation functional thin-film and soft material systems.

Overall, the contributions in this Special Issue show that gel-based materials can no longer be viewed as passive matrices, but rather as programmable functional systems whose performance across a broad range of applications is governed by the interplay between structure, composition, and processing.

Despite these advances, challenges remain. Further studies are needed for a better understanding of gelation dynamics in thin films, the influence of drying and post-treatment on stability, and the relationship between hierarchical structure and performance of devices. In addition, improved reproducibility and integration of gel-based materials into practical platforms remain important directions for future research.
